# The Rationale for Discarding Blood and Its Components in a Tertiary Care Hospital Blood Bank in North Karnataka

**DOI:** 10.7759/cureus.31112

**Published:** 2022-11-04

**Authors:** Keshav R Kulkarni, Pavan Kulkarni, Uma Jamkhandi

**Affiliations:** 1 Department of Pathology, S Nijalingappa Medical College and HSK (Hanagal Shree Kumareshwar) Hospital and Research Centre, Bagalkote, IND

**Keywords:** blood transfusion services, audit, discard rate, blood components, whole blood

## Abstract

Background

Human blood is an essential human element for which no substitute has yet been discovered. This study aims to determine the causes of discarding blood and its components.

Material and methods

A retrospective study of three years (January 1, 2018, to December 31, 2020) was performed in a tertiary care hospital blood bank. Data were retrieved from the various registers and analysed.

Results

During this study, a total of 3280 units, 1868 units, and 486 units of whole blood were collected in 2018, 2019, and 2020, respectively. It was observed that the discard rate of whole blood was 9.48%, 17.23%, and 43% in 2018, 2019, and 2020, respectively. The discard rate for components varied, such as for packed red blood cells (PRBC), it was 1.76% in 2018, 1.73% in 2019, and 3.03% in 2020, for fresh frozen plasma (FFP), it was 4.08% in 2018, 4.36% in 2019, and 2.20% in 2020, and for platelets, it was 43.08% in 2018, 31.56% in 2019, and 45.03% in 2020. A total of 311, 322, and 209 units of whole blood were discarded in 2018, 2019, and 2020, respectively. The total whole blood and blood components were discarded for various reasons such as undiagnosed sterility (HIV, hepatitis B surface antigen, hepatitis C virus, Venereal Disease Research Laboratory), quality control, underweight, hemolysis unit, expiry, and polycythemia.

Conclusion

The wastage of stored blood and its components is inevitable. Still, it can be minimised by optimum utilisation and implementation of blood transfusion services (BTS) along with the education and training of blood bank staff. There are various reasons for the wastage of blood and its components, such as unscreened transfusion-transmitted diseases, sterility, storage, less bleeding, expiry, hemolysis samples and polycythemia. Self-regular audits, coordination between hospital and blood bank staff, proper storage and handling of blood units, strict donor selection and deferral criteria, along with appropriate history taking, will help minimise the wastage of blood or its components.

## Introduction

Human blood is an essential element of human life and there is no artificial substitute for blood that has been discovered yet. As its availability is limited, blood transfusion services (BTS) play a vital role in any national health service delivery system. Thus, the availability of safe and adequate blood saves lives and prevents the wastage of this valuable resource. Human blood is categorised as a “drug” under section 3(b) of the Drugs and Cosmetics Act 1940 in India [1]. It has been estimated that every two seconds, someone across the world requires blood [2-4]. For many chronic illnesses like beta-thalassemia major, chemotherapy requires continued blood transfusions from healthy donors. The rate of discarded blood components or “wastage rate” is one of the 10 quality indicators recommended by the National Accreditation Board for Hospitals & Healthcare Providers (NABH), India [5,6]. This study aims to figure out the various causes of discarding blood and its components, so that blood and its component preparation, and use of blood can be optimised through proper education and training of staff.

## Materials and methods

This is a retrospective study conducted in the Department of Pathology of S. Nijalingappa Medical College and HSK Hospital and Research Center, Bagalkot, Karnataka, India, to find out the various causes of the discarding of blood and its components in the blood bank of a tertiary care hospital for three years from January 1, 2018, to December 31, 2020. The selection of blood donation was made according to the World Health Organisation (WHO) selection criteria. Data were retrieved from various registers like the donor register, discard register, transfusion-transmitted diseases (TTDs) register, and components preparation register. The rationale for discarding blood and its components was analysed.

Components preparations and discard of blood bags were done according to standard operating procedures and guidelines laid by National Aids Control Organisation (NACO), India, and our blood bank guidelines. The present study includes blood units discarded for various reasons like TTDs, seroreactivity, expired components, less bleed, breakage, clotted bag, hemolysed samples, and units sent for quality checks (QC). At the same time, no exclusion criteria were followed for the selection of the samples.

All data were analysed using IBM SPSS Statistics for Windows, Version 26.0 (Released 2019; IBM Corp., Armonk, New York, United States). All categorical data were represented as frequency and percentages.

## Results

A total of 3280 units, 1868 units, and 486 units of whole blood were collected in 2018, 2019, and 2020, respectively. The various components such as packed red blood cells (PRBC), fresh frozen plasma (FFP), and platelets were prepared. In 2018, 3946 units of PRBC and FFP, along with 1408 units of platelets, were prepared. In 2019, 3577 units of PRBC and FFP and 1302 units of platelets were prepared. Similarly, in 2020, 2772 units of PRBC and FFP, along with 635 units of platelets, were prepared (Table 1).

**Table 1:** Total units of the different components of blood collected PRBC: packed red blood cells; FFP: fresh frozen plasma

	2018	2019	2020
Whole blood	3280	1868	486
PRBC	3946	3577	2772
FFP	3946	3577	2772
Platelet	1408	1302	635

It was observed that the discard rate of whole blood was 9.48%, 17.23%, and 43% in 2018, 2019, and 2020, respectively. The discard rate for components varied such as for PRBC it was 1.76% in 2018, 1.73% in 2019, and 3.03% in 2020, for FFP it was 4.08% in 2018, 4.36% in 2019, and 2.20% in 2020, and for platelet it was 43.08% in 2018, 31.56% in 2019, and 45.03% in 2020 (Table 2).

**Table 2:** Discard rate of whole blood and its components PRBC: packed red blood cells; FFP: fresh frozen plasma

Blood components	YEAR		
	2018	2019	2020
	No. of units discarded(%)	No. of units discarded(%)	No. of units discarded(%)
Whole blood	311(9.48)	322(17.23)	209(43)
PRBC	66(1.67)	62(1.73)	84(3.03)
FFP	161(4.08)	156(4.36)	61(2.20)
Platelet	617(43.82)	411(31.56)	286(45.03)
Total	1155(59.05)	951(54.88)	640(91.06)

A total of 311, 322 and 209 units of whole blood were discarded in 2018, 2019, and 2020, respectively. The total whole blood discarded due to various reasons such as undiagnosed sterility (HIV, hepatitis B surface antigen (HBsAg), hepatitis C virus (HCV), Venereal Disease Research Laboratory (VDRL), India), quality control, underweight, hemolysed unit, expiry, and polycythemia and average discard rates are shown in Table 3.

**Table 3:** Year-wise causes of wastage of whole blood HIV: human immunodeficiency virus; HBsAg: hepatitis B surface antigen; HCV: hepatitis C virus; VDRL: Venereal Disease Research Laboratory, India

Wastage causes		YEAR		
		2018	2019	2020
		No. of units (%)	No. of units (%)	No. of units (%)
Seropositivity for transfusion-transmitted diseases	HIV	21(6.75)	10(3.10)	04(1.91)
	HBsAg	181(58.19)	147(45.6)	83(39.71)
	HCV	08(2.57)	10(3.10)	07(3.34)
	VDRL	00(00)	00(00)	00(00)
Storage (QC)		63(20.25)	67(1.55)	55(26.31)
Less bleed (underweight)		05(1.60)	05(1.55)	04(1.91)
Expiry		17(5.46)	44(13.66)	51(24.4)
Hemolysed sample		08(2.57)	23(7.14)	01(0.47)
Polycythemia		08(2.57)	16(4.96)	04(1.91)
Total		311	322	209

Similarly, a total of 844, 629, and 431 units of blood components (PRBC, FFP, platelets) were discarded in 2018, 2019, and 2020, respectively. The discard rate of blood components was highest and lowest in 2018 and 2020, respectively. Among all three components, platelets have a high discard rate for all three years (Table 4).

**Table 4:** Year-wise causes of blood components discard PRBC: packed red blood cells; FFP: fresh frozen plasma

	YEAR								
Reasons	2018			2019			2020		
	No. of units (%)			No. of units (%)			No. of units (%)		
	PRBC	FFP	Platelets	PRBC	FFP	Platelets	PRBC	FFP	Platelets
Seropositivity	35	25	58	35	26	38	44	24	44
Less bleed	03	00	00	04	00	00	04	00	17
Expiry	12	57	559	10	00	373	35	00	224
Hemolysed	14	06	00	13	06	00	00	00	00
breakage	02	73	00	00	124	00	01	37	01
Total	66	161	617	62	156	411	84	61	286

## Discussion

Despite advancements in modern technology, there is no substitute for blood discovered yet. Thus, BTS plays a vital role in modern day-to-day clinical practice. Furthermore, blood and its components provide unique and lifesaving therapeutic benefits to patients [7-9]. The need for blood and its components is increasing due to advanced improvement and accurate diagnosis of complex diseases requiring repeated transfusion. 

Owing to resource constraints, it is not always possible for blood or its components to reach the patient at the right time. The primary concern is for safe, effective, quality blood to be available to patients when needed. Thus, proper blood management at the blood bank and training and optimised staff education will prevent the unnecessary wastage of blood and its products [7].

The present study showed that 311 (9.48%) of the 3280 units, 322 (17.23%) of the 1868 units, and 209 (43%) of the 486 units of whole blood were discarded in 2018, 2019, and 2020, respectively. The average discard rate for whole blood in the present study was 23.23%, which was higher than in studies by Suresh et al. (5.7%), Bobde et al. (6.63%), Sharma et al. (4.46%), and Kanani et al. (3.5%) [2,10,11,7].

The various reasons for the discard of blood are seropositive for transfusion-transmissible infections (TTI) like HIV, HBsAg, HCV, VDRL, India, testing for syphilis, sterility (quality control), less bleeding (underweight), expiry, hemolysed sample, and polycythemia [12-15]. Our study showed seropositivity for TTI as the most common cause of wastage of whole blood and its components (HBsAg>HIV>HCV> VDRL), followed by sterility, expiry, hemolysed sample, polycythemia, and less bleeding. It is similar to a study conducted by Suresh et al. [2]. However, it can be minimised by proper history taking, strict adherence to donor selection criteria, and creating a donor database. Another reason for discarding whole blood in the present study was sterility check or quality control, which is carried out as a part of the Drugs and Cosmetics Act of 1940, India [1]. It has to be followed in all blood banks. Hence, this could not be prevented.

The limitation of our study was less bleeding/underweight blood bags because of phlebotomy failure like the collapse of veins, uneasiness, vomiting, perspiration, hematoma formation, and fainting during donation. Such blood bags are unsuitable for transfusion as there is a mismatch between the amount of blood collected and the anticoagulant used in the bag. Such collection usually happens in blood donation camps. It can be prevented by the proper selection of healthy donors, motivation of donors, and adequate training of phlebotomy staff [12]. In the present study, the most common cause of discard of FFP was breakage, followed by seropositivity for TTI, expiry, and other reasons. It happens because of inappropriate handling of these component bags during processing/centrifugation, and breakage of FFP bags was mainly observed during the removal of bags from the deep freezer before thawing [14]. Leakage of blood bags can be prevented by proper visual inspection of blood bags during centrifugation. Breakage of FFP bags can be prevented by proper handling and covering FFP bags in protective containers [8]. Our study showed expiry was the most common cause of discarding platelets, followed by seropositivity, less bleeding, and breakage. The shelf life of platelets is five days. Thus, their chance of expiry was highest due to the non-utilisation of blood products [15]. In the present study, the discard of platelets was highest among the components prepared. Its wastage can be minimised by optimal preparation according to the clinical requirement and increased use of the apheresis technique.

## Conclusions

The wastage of blood and its components is inevitable, but it can be minimised by optimum utilisation and implementation of BTS along with the education and training of blood bank staff. There are various reasons for the wastage of blood and its components, such as sterility, storage, less bleeding, expiry, hemolysed sample, and polycythemia. Self-regular audits, coordination between hospital and blood bank staff, proper storage and handling of blood units, strict donor selection and deferral criteria, along with proper history taking, will help minimise the wastage of blood and its components.
